# Desk-top computer as interface and terminal in the clinical laboratory

**DOI:** 10.1155/S1463924682000431

**Published:** 1982

**Authors:** J. I. Dydula, O. S. Lauritsen, R. Dybkaer

**Affiliations:** Department of Clinical Chemistry Frederiksberg Hospital Nordre Fasanvej 59 Copenhagen F DK2000 Denmark


Journal of Automatic Chemistry, Volume 4, Number 4 (October–December 1982), pages 174–175

### Desk-top computer as interface and terminal in the clinical laboratory

J. I. Dydula, O. S. Lauritsen and R. Dybkaer

*Department of Clinical Chemistry, Frederiksberg Hospital, Nordre Fasanvej 59, DK2000 Copenhagen F*

### Introduction

During the last two decades computer technology has been introduced in many hospital laboratories according to one of two main strategies of laboratory data acquisition and management: the 'turn-key' system and the 'do-it-yourself' system. Drawbacks of the former include its poor ability to solve unforeseen problems or to adapt to changing work organization, and its total dependency on supplier support. A large do-it-yourself system, on the other hand, is very demanding of personnel and time.

About 10 years ago the authors' laboratory had problems with a turn-key central computer system and had to supplement it with self-designed interfaces and terminals [1]. Then, in conjunction with a change of central computer, the authors' sought another approach and found that progress in microprocessors and microcomputers now permits a combination of the two strategies [2].

Based on previous experience and urged by a recent editorial [3], the authors discuss some interface problems, the interesting possibilities of the desk-top or 'personal' computer, and a set of suggested requirements.

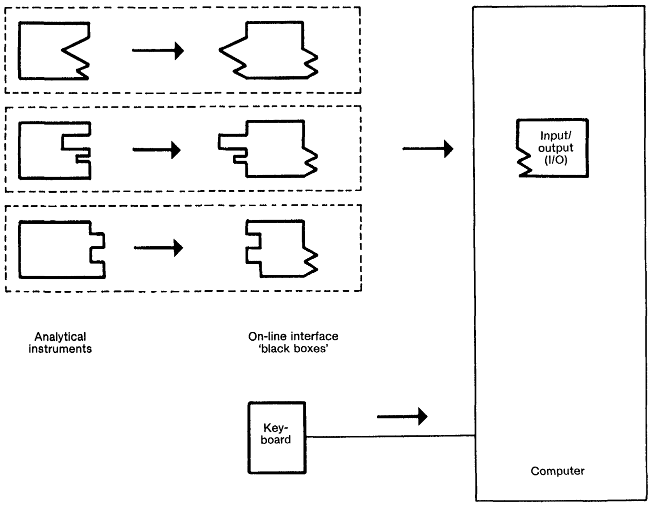

*Figure 1. Analytical instruments connected to a central computer through different dedicated interface devices ('black boxes').*

### Interface problems

It is characteristic of the larger hospital laboratory that results derived from patient specimens are generated according to many different analytical principles and by procedures ranging from a simple manual method to a highly automated 'multichannel' system.

Modern analysers are equipped with data-output devices, but as these occur in many configurations they often do not fit into the input devices of large computers. This incompatibility has led to the construction of numerous dedicated interface devices (see figure 1). Such 'black boxes', however, are rigid and costly, both in the turn-key and do-it-yourself systems.

### Advantages of the desk-top computer

Typically, the desk-top computer may function both as an interface and as a terminal with several tasks, permitting two-way communication between analyser, technician, and central computer. Advantages are: a *flexible interface* for data capture from different types of analysers and data transfer to the large computer. *Introduction of specimen identification* into the computer in a variety of ways, depending on the the type of work station. *Convenient error correction* of ID numbers and results. *Quality control at work station* of each result and series, leading to increased technician involvement and responsibility. *Back-up facilities* during computer break-down with data storage for several days in an external memory or as hard-copy print-out or recording. *Software changes* may be made by the user, adapting to changed working conditions.

### Requirements of the clinical laboratory's desk-top computer

Faced with a jungle of different models, the explorer may be helped by some suggestions for useful characteristics [4].

### *Input/output (I/O) facilities*

(1) A minimum of four users' accessory expansion slots.
(2) A broad selection of I/O print cards allowing communication with a range of instruments and computer I/O standards. One of the print cards should be an A/D converter with a minimum accuracy of nine bits.

### *Hardware features*

(1) A TV monitor with a minimum of 24 lines.
(2) A numerical keyboard supplemented with at least eight function keys.
(3) One or two disc units.
(4) A printer with at least 80 character-lines, and preferably one which uses ordinary paper.
(5) Separate housing of these units for easy error detection and replacement.

### *Memory*

(1) A minimum of 48 kilobytes of random access memory (RAM). This extra expense amounts to only a small fraction of the total price.

 0142–0453/82/0404 0174 $05·00 © 1982 Taylor & Francis Ltd

(2) An external memory of more than 200 kilobytes either as tape or, better, as disc units to obtain shorter access time.

### *Programming language*

The language should be easy to learn and use by novices to data processing; BASIC is a good example.

### *Program library*

(1) A broad selection of standard programs should be available in the fields of mathematics, statistics, quality assessment, and graphics. The descriptions should not assume previous programming experience.
(2) An easily manipulated graphical display of relations on the TV monitor is desirable; its accuracy, as a rule, is of secondary importance in the clinical laboratory.

### *Instructions*

(1) A comprehensive user's manual in ordinary language is needed.
(2) A comprehensive test and demonstration program on tape or disc for checking and trouble-shooting is helpful.

### *Source and support*

(1) The manufacturer or distributor should be reliable and capable of supplying continued system assistance and service; also, he should be able to ensure consultants' support. Otherwise, the desk-top computer could become a liability.
(2) It is very important to have consultants' help in developing or modifying I/O print cards and the necessary assembler subroutines.

### *Availability*

A very popular model, sold in large numbers, makes regional and international exchange of user-made programs and experience more likely.

### *Uniformity*

The use of essentially one type of desk-top computer in a laboratory facilitates programming, maintenance, service, and teaching—and reduces the cost.

### Recent implementation

The replacement of an old on-line haematology multichannel analyser with a new, and larger, Coulter Counter Model S-Plus one year before changing the central computer from an IBM System 1800 to an IBM System 4300 meant that a flexible system was needed in the authors' laboratory. Considering the requirements listed above, an Apple II microcomputer was chosen (see figure 2) and a keyboard with numerical and function keys added.

The Coulter Counter Model S-Plus is an automated multichannel analyser for particle counting of different types of blood cells, size distribution, and spectrometry of haemoglobin. Up to 12 results on each specimen are obtained in 50 s.

The following sequence of events was chosen. Blood is held to the suction device, which is activated by a push-button. The ID number is visually read off the label of the hand-held specimen tube and introduced via the keyboard. After about 50 s of analysis time, the results are automatically transferred to the Apple II, displayed on the TV monitor for inspection, and printed, before being released by the technician for transmission to the central computer. The next specimen is aspirated, the ID number is introduced, and during the time of analysis the ID

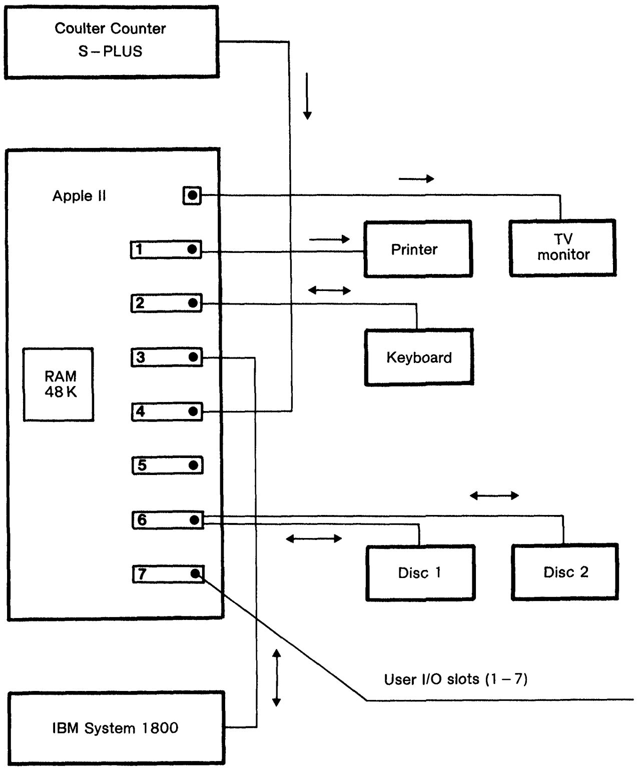

*Figure 2. Interfacing a Coulter Counter Model S-Plus with an IBM System 1800 computer using an Apple II desk-top computer.*

number and results of the previous specimen are stored on disc 'D2' for back-up and evaluation of statistics. Programs for data acquisition, processing, transmission, statistics, TV monitoring, printing etc. are stored on disc 'D1'.

Further interfacing has been achieved for a Technicon SMA IIC eight-channel pressure flow analyser and a Roche Cobas Bio centrifugal analyser. It is planned to continue with a Technicon SMA 6/60 six-channel analyser, a Coulter Counter Model S, and a Technicon AutoAnalyzer II single-channel pressure flow analyser.

### References

1. Dydula, J. I. and Lauritsen, O. S., *Proceedings 2nd ISPRA Nuclear Electronics Symposium* (held in Stresa, Italy, from 20–23 May 1975, published by Commission of the European Communities, Luxembourg, 1975), p. 241.
2. Liscouski, J. G., *Analytical Chemistry*, **54** (1982), 849A.
3. Arndt, R. W., *Journal of Automatic Chemistry*, **3** (1981), 167.
4. Broadbridge, R. and Heslop, J. A., *International Laboratory*, **12**, 3 (1982), 72.

